# Expression characteristics and detection value of Ki67, estrogen receptor (ER), progesterone receptor (PR), human epidermal growth factor receptor 2 (HER-2) in patients with breast cancer

**DOI:** 10.12669/pjms.42.1.13351

**Published:** 2026-01

**Authors:** Weiwei Qian, Yi Ni, Zihan Wang, Fei Yin

**Affiliations:** 1 Weiwei Qian, Department of Thyroid and Breast Surgery, Affiliated Nantong Hospital 3 of Nantong University, Nantong, Jiangsu Province 226001, P.R. China, Department of Thyroid and Breast Surgery, Nantong Third People’s Hospital, Nantong, Jiangsu Province 226001, P.R. China; 2 Yi Ni, Department of Thyroid and Breast Surgery, Affiliated Nantong Hospital 3 of Nantong University, Nantong, Jiangsu Province 226001, P.R. China, Department of Thyroid and Breast Surgery, Nantong Third People’s Hospital, Nantong, Jiangsu Province 226001, P.R. China; 3 Zihan Wang Department of Surgery, School of Medicine, Nantong University, Nantong, Jiangsu Province 226000, P.R. China, Department of Thyroid and Breast Surgery, Affiliated Nantong Hospital 3 of Nantong University, Nantong, Jiangsu Province 226001, P.R. China, Department of Thyroid and Breast Surgery, Nantong Third People’s Hospital, Nantong, Jiangsu Province 226001, P.R. China; 4 Fei Yin, Department of Thyroid and Breast Surgery, Affiliated Nantong Hospital 3 of Nantong University, Nantong, Jiangsu Province 226001, P.R. China, Department of Thyroid and Breast Surgery, Nantong Third People’s Hospital, Nantong, Jiangsu Province 226001, P.R. China

**Keywords:** Breast cancer, Estrogen Receptor, Progesterone Receptor, Human epidermal growth factor receptor 2

## Abstract

**Objective:**

To investigate the expression characteristics of Ki-67, estrogen receptor (ER), progesterone receptor (PR), and human epidermal growth factor receptor 2 (HER-2) in breast cancer (BC) patients and to evaluate their associations with lymph node metastasis and clinical disease stage.

**Methodology:**

The clinicopathological data of 122 patients with BC admitted to Affiliated Nantong Hospital 3^rd^ of Nantong University from April 2020 to November 2023 were analyzed retrospectively. The immunohistochemical expression of Ki67, ER, PR (labeling indices, %) and HER-2 (IHC score, 0–3+) was compared between groups stratified by lymph node metastasis status and clinical disease stage, and correlations were assessed using Spearman rank analysis.

**Results:**

Patients with lymph node metastasis had a significantly higher Ki67 labeling index and significantly lower ER and PR labeling indices than those without lymph node metastasis (all *P*<0.05), whereas HER-2 IHC scores did not differ significantly between the two groups (*P*>0.05). Across clinical stages, the Ki67 labeling index increased progressively from Stage I to Stage IV, whereas ER and PR labeling indices decreased correspondingly, with all pairwise comparisons between adjacent stages remaining statistically significant (*P*<0.05). Spearman analysis showed that the Ki67 labeling index was positively correlated with lymph node metastasis and clinical disease stage, whereas ER and PR labeling indices were negatively correlated with both parameters (*P*<0.05). In contrast, HER-2 IHC score showed no significant correlation with lymph node status or clinical stage (*P*>0.05).

**Conclusions:**

BC patients exhibit characteristic alterations in Ki67, ER, and PR immunohistochemical expression, and the degree of Ki67 increase together with ER and PR decrease is closely associated with lymph node metastasis and clinical disease stage. These findings suggest that combined assessment of these biomarkers may be useful for risk stratification and staging in breast cancer.

## INTRODUCTION

Breast cancer (BC) is caused by the uncontrolled proliferation of breast epithelial cells under the action of various carcinogens.^1^,^2^ It is considered the most common malignant tumor in women, is highly metastatic, and is associated with high mortality rates.^3^,^4^ Early and accurate diagnosis and evaluation of BC are of great significance, as they can guide timely clinical implementation of targeted treatments.^2^-^4^

BC is a hormone-dependent tumor.^4^ Numerous studies have identified estrogen receptor (ER), progesterone receptor (PR), and human epidermal growth factor receptor 2 (HER2) as primary BC markers that allow for decision-making in the metastatic setting.^5^-^8^ Both estrogen receptor and progesterone receptor play essential roles in cellular inflammatory responses.^9^,^10^ Some studies have shown that the positive expression rate of PR can reach 70.0%.^10^,^11^ The positive expression rate of ER is relatively low in healthy breast tissue, but can reach 50.0% to 70.0% in breast malignant tumors.^11^,^12^ HER-2, located on chromosome 17q21, can regulate cell proliferation and growth, and has tumor transformation activity.^13^ Antigen Ki-67 (Ki-67), another known marker of cancer proliferation, has been shown to have prognostic value in invasive BC.^14^,^15^

Although numerous studies have examined Ki67, ER, PR, and HER-2 as prognostic or predictive biomarkers in breast cancer,^5^,^7^,^8^,^12^,^15^ most research has focused on individual markers or limited clinical endpoints. Few studies have conducted an integrated analysis of these four markers within the same patient cohort to assess their relationship with both lymph node metastasis and tumor stage.^14^ This study aimed to address this gap by evaluating the expression patterns of Ki67, ER, PR, and HER-2 and their associations with key pathological features. We hypothesize that abnormal expression of these biomarkers is significantly correlated with disease progression and nodal involvement, and that they may hold potential clinical value for diagnosis and staging.^1^,^15^ The novelty of our study lies in its comprehensive design: using a consistent dataset, we systematically analyze four key biomarkers across multiple disease stages and nodal statuses, providing a more holistic understanding of their diagnostic utility in breast cancer.^13^

## METHODOLOGY

Clinical and pathological data of 122 patients with BC admitted to Affiliated Nantong Hospital 3^rd^ of Nantong University from April 2020 to November 2023 were retrospectively selected. ***Ethical Approval:*** The ethics committee of our hospital approved the study with the number EK2024041, Date: October 29^th^ 2024.

### Inclusion criteria:

- Meet the diagnostic criteria for BC.^1^
- BC confirmed through pathological examination.
- No intervention such as immunotherapy, chemotherapy, or radiotherapy before the examination.
- Unilateral onset.
- First onset of illness.
- Complete clinical data.

### Exclusion criteria:

- Individuals with other malignant tumors.
- Individuals with coagulation dysfunction.
- Individuals with autoimmune or hematological diseases.
- Secondary BC.
- Individuals with other breast diseases.
- Patients with distant metastasis of the lesion.
- Recurrent BC.
- Individuals with a history of surgical treatment for breast related diseases.

### Collected data:

- Patient baseline data, including age, body mass index (BMI), lymph node metastasis, and disease staging.
- Immunohistochemical staining for Ki67, ER, PR, and HER-2 was performed in the pathology department as part of the routine diagnostic work-up, including sampling of the tumor and surrounding tissues, other breast tissue, axillary and mammary gland lymph nodes, the nipple, and the deep margins. All patients underwent complete lymph node sampling to minimize false-negative results. Larger volumes were subjected to incision and embedding procedures. Each embedding cassette contained no more than five lymph nodes. For larger specimens, separate embedding and sectioning were performed. Embedded tissues were cut into three μm-thick sections. The sections were treated to inhibit endogenous peroxidase activity using 3% hydrogen peroxide for approximately 10 minutes, and then washed with PBS. Antigen retrieval was performed according to the requirements of the primary antibody. After the retrieval, sections were incubated with the respective primary antibody for 35 minutes, washed, and incubated with a secondary antibody for approximately 20 minutes. The 3’3’diaminobenzidine (DAB) chromogen was used to visualize the signal, and the sections were observed under a microscope. The reaction was stopped with water, and nuclear counterstaining was performed. For Ki67, ER, and PR, only nuclear staining in invasive tumor cells was considered specific. For each case, at least 500 tumor cells were counted in five randomly selected high-power fields (×400). The Ki67 index was defined as the percentage of tumor cell nuclei showing unequivocal positive staining and was recorded as a continuous variable (%). ER and PR expression were scored as the percentage of tumor cell nuclei with any specific staining, irrespective of intensity, and were also recorded as continuous variables (%). According to the ASCO/CAP guidelines, ER and PR status were considered positive when ≥1% of tumor cell nuclei were immunoreactive.

### HER-2 interpretation criteria:

Cases with no staining or ≤10% of infiltrating cancer cells showing weak and incomplete membrane staining were scored as 0. Cases with ≥10% of infiltrating cancer cells showing weak and incomplete membrane staining were scored as 1+ (+). Cases with ≤10% of infiltrating cancer cells showing strong and complete membrane staining, or >10% of cells showing incomplete and/or weak to moderate membrane staining, were scored as 2+ (++). Cases with >10% of infiltrating cancer cells displaying uniform, complete, and strong circumferential membrane staining were scored as 3+ (+++).

### Statistical analysis:

Data was analyzed using SPSS 26.0. Use *(X̄±S)* to describe quantitative data. Independent-sample t-tests and one-way ANOVA were used for intergroup comparisons. Levene’s test was performed to verify the homogeneity of variances before applying ANOVA. For post hoc pairwise comparisons, the LSD (Least Significant Difference) test was used, as all variables satisfied the equal variances assumption (*P*>0.05). LSD was considered appropriate given the limited number of comparison groups and the goal of maintaining sensitivity without overcorrecting Type I error. Counting data was described as frequency and composition ratio (%), and the chi-square test was used. The correlations of the Ki67, ER, and PR labeling indices (expressed as the percentage of positively stained tumor cell nuclei) and the HER-2 immunohistochemical scores (0–3+) with lymph node metastasis and clinical stage of BC were analyzed using Spearman’s rank correlation. *P*<0.05 indicated a statistically significant difference.

## RESULTS

This study included 122 BC patients. As shown in the Table-I patient’s age ranged from 39 to 78 years, with an average of 58.39 ± 13.62 years. The BMI ranged from 18.2 to 27.6 kg/m2, with an average of 22.81 ± 3.64 kg/m2. There were 56 cases with lymph node metastasis and 66 cases without metastasis. In terms of the disease staging, 32 cases were in Stage-I, 69 cases in Stage-II, 18 cases in Stage-III, and three cases in Stage-IV.

**Table-I:** Comparison of Ki67, ER, PR, and HER-2 levels in patients with different lymph node metastases.

Lymph node metastasis situation	n	Ki67 labeling index (%)	ER labeling index (%)	PR labeling index (%)	HER-2 IHC score (0-3)
With metastasis	56	40.0 ± 12.0	65.0 ± 20.0	40.0 ± 18.0	1.5 ± 1.0
Without metastasis	66	25.0 ± 10.0	80.0 ± 15.0	60.0 ± 20.0	1.3 ± 1.1
*t*		7.42	4.62	5.81	1.05
*P*		＜0.001	＜0.001	＜0.001	0.295

***Note:*** Data are presented as mean ± standard deviation. Ki67, ER, and PR are expressed as labeling indices, defined as the percentage of positively stained tumor cell nuclei (%). HER-2 is expressed as the immunohistochemical score (0–3+). Independent-samples t-tests were used for between-group comparisons.

Patients with lymph node metastasis showed a significantly higher Ki67 labeling index and significantly lower ER and PR labeling indices compared with those without lymph node metastasis (all *P*<0.05). In contrast, the HER-2 immunohistochemical score did not differ significantly between patients with and without lymph node metastasis (*P*>0.05) (Table-I).

As shown in Table-II, the Ki67, ER, and PR labeling indices differed significantly among patients with different clinical stages (all *P*<0.05), whereas the HER-2 immunohistochemical score did not show a statistically significant difference across stages (*P*>0.05). Post hoc comparisons indicated that Stage-II patients had a higher Ki67 labeling index and lower ER and PR labeling indices than Stage I patients (all *P*<0.05). Similarly, Stage-III patients exhibited a further increase in the Ki67 labeling index and a further decrease in ER and PR labeling indices compared with Stage-II patients (all *P*<0.05). Stage IV patients showed markedly elevated Ki67 labeling indices accompanied by substantially lower ER and PR expression compared with Stage-III patients (all *P*<0.05). Consistent with the overall analysis, pairwise comparisons did not reveal statistically significant differences in HER-2 immunohistochemical scores between individual clinical stages (*P*>0.05).

**Table-II:** Comparison of Ki67, ER, PR, and HER-2 levels in patients with different disease stages.

Disease Staging	n	Ki67 labeling index (%)	ER labeling index (%)	PR labeling index (%)	HER-2 IHC score (0–3+)
Ⅰ	32	22.0 ± 6.0	56.9±9.4	50.0±5.3	1.2±1.0
Ⅱ	69	30.0 ± 8.0^a^	55.0 ± 15.0^a^	45.0 ± 20.0^a^	1.4 ± 1.0^a^
Ⅲ	18	38.0 ± 10.0^ab^	50.0 ± 15.0^ab^	30.0 ± 18.0^ab^	1.6 ± 1.1^ab^
Ⅳ	3	45.0 ± 12.0^abc^	40.0 ± 10.0^bc^	10.0 ± 8.0^abc^	2.1 ± 0.8^abc^
*F*		20.25	18.02	24.14	2.36
*P*		＜0.001	＜0.001	＜0.001	0.075

***Note:*** Data are presented as mean ± standard deviation. Ki67, ER, and PR are expressed as labeling indices (% positive tumor cell nuclei). HER-2 is expressed as the immunohistochemical score (0–3+). One-way ANOVA was used to compare groups, followed by LSD t-tests for pairwise comparisons.

a P<0.05 vs Stage I; ^b^P<0.05 vs Stage-II; ^c^P<0.05 vs Stage-III.

Spearman rank correlation analysis confirmed that the Ki67 labeling index was positively correlated with lymph node metastasis and clinical disease stage in BC patients (*P*<0.05). In contrast, the ER and PR labeling indices were negatively correlated with lymph node metastasis and clinical stage (*P*<0.05). The HER-2 immunohistochemical score showed no significant correlation with either lymph node metastasis status or clinical disease stage (*P*>0.05) (Table-III).

**Table-III:** Correlation analysis of each index level with lymph node metastasis and disease stage of BC.

Pathological features	Ki67 labeling index		ER labeling index		PR labeling index		HER-2 IHC score	
	r	P	r	P	r	P	r	P
Lymph node metastasis situation	0.38	＜0.001	-0.50	＜0.001	-0.38	＜0.001	0.14	>0.05
Disease Staging	0.42	＜0.001	-0.42	＜0.001	-0.47	＜0.001	0.161	>0.05

***Note:*** Ki67, ER, and PR were analyzed as continuous variables based on their labeling indices (% positive tumor cell nuclei). HER-2 was analyzed as an ordinal variable using the IHC score (0–3+). Spearman rank correlation was used.

## DISCUSSION

The results of this study showed significant differences in the expression of Ki67, ER, and PR in BC. Changes in these immunohistochemical indices were closely associated with lymph node metastasis and clinical disease stage. Zhang et al.^16^ showed that the median progression-free survival period (25 months) of BC patients with high levels of ER and PR after effective treatment was longer than that of patients with low levels of ER and PR. Additionally, there was a significant difference in clinical benefit rate (80.9% vs. 55.6). Studies have showed that PR expression is induced by the estrogen-ER interaction,^17^,^18^ and can regulate the growth of BC cell lines, tumor cell infiltration, and metastasis.^19^,^20^ Hussein et al.^21^ examined the pathological tissue sections of BC patients and showed that BRCA1-negative patients showed negative expression of ER, PR, and Her2/neu.

Wang et al.^22^ confirmed that PR- and ER-positive tumor cells respond to signals from progesterone and estrogen, and that inhibiting tumor cell proliferation using progesterone and estrogen may exert targeted effects. With the progression of BC, the original biological characteristics of cells are gradually lost, and the expression of PR and ER is reduced,^23^ which is further supported by the results of this study.

A study by de Gregorio et al.^24^ found that Ki67 expression in BC patients was abnormally high, and the degree of increase positively correlated with lymph node positivity and clinical stage. Song et al.^25^ also demonstrated considerably higher expression and protein levels of Ki67 in the tumor tissue of BC patients compared to the adjacent tissues. The degree of expression was closely related to lymph node metastasis, disease staging, tissue grading, etc. Ki67 is a nuclear antigen closely related to cell mitosis that gradually forms in the middle and late stages of the G1 cell cycle and reaches its peak in the M phase.^26^ Due to the higher proliferation and division rates of cancer cells, Ki67 expression is significantly higher in cancerous tissues with poorly differentiated tumor cells compared to normal tissue.^27^,^28^

Moreover, higher Ki67 expression directly correlates with higher proliferation activity and invasiveness of BC cells, therefore, positively correlating with the disease stage.^25^,^27^,^28^ The study by Shet et al. 29 also pointed out that Ki67 can be used as a marker for the severity, progression, and prognosis of cancer, which is in line with the results of this study. In addition, this study found no significant differences in HER-2 immunohistochemical scores among patients with different lymph node metastasis statuses and disease stages. However, studies have showed that HER-2 can affect the expression of tumor suppressor genes, activate tumor necrosis factor-α, and accelerate tumor cell proliferation. Furthermore, the expression of HER-2 is closely related to pathological features such as disease staging.^30^-^32^ The observed differences between the previous reports and this study may be related to the variability in the severity of the selected cases as well as regional differences.

Beyond its prognostic association, Ki67 also holds significant clinical utility in treatment decision-making, as emphasized in recent international guidelines. The St. Gallen International Consensus (2021), together with the NCCN and ESMO Breast Cancer Guidelines, recommends using Ki67 to help distinguish Luminal A from Luminal B subtypes and to guide decisions regarding adjuvant chemotherapy in hormone receptor–positive patients.^1^,^33^,^34^ In our study, the Ki67 labeling index increased consistently with both advancing clinical stage and the presence of lymph node metastasis, reinforcing its role as a high-risk marker in breast cancer. This pattern supports its biological link to tumor cell proliferation and poorer prognosis, and strengthens the rationale for using Ki67-based risk stratification to determine the need and intensity of adjuvant systemic therapy in selected patients. Although our dataset did not include follow-up or treatment outcome information, future prospective, therapy-guided studies are warranted to further clarify the predictive value of Ki67 for treatment response and recurrence risk, particularly in early-stage and hormone receptor–positive breast cancer.

Several factors may help explain the lack of statistical association between HER-2 expression and disease stage or lymph node status in our findings. First, the overall proportion of HER-2 3+ positive cases in our sample was relatively low, which may have weakened statistical power. Second, due to the retrospective nature of this study, HER-2 2+ equivocal cases were not routinely validated with fluorescence in situ hybridization (FISH), potentially resulting in underestimation or misclassification of HER-2 positivity. This has been clearly acknowledged as a methodological limitation. Third, HER-2 scoring in immunohistochemistry is susceptible to observer variability, particularly for borderline cases, which may have affected classification accuracy. Moreover, recent literature suggests that HER-2 expression does not consistently correlate with standard clinicopathological features, which may reflect the molecular heterogeneity of breast cancer.^13^,^32^ These findings underscore the importance of FISH confirmation, HER-2 evaluation standardization, and the incorporation of molecular subtyping in future studies.

In summary, this study provides a comprehensive analysis of four key breast cancer biomarkers—Ki67, ER, PR, and HER-2—across different disease stages and lymph node statuses within the same patient cohort. This integrated approach highlights the distinct expression patterns of these markers as breast cancer progresses, offering new insights beyond studies focusing on single indicators. Clinically, our findings demonstrate that the Ki67 labeling index increases, whereas ER and PR expression decreases with disease progression, supporting their use not only for diagnostic work-up but also for treatment stratification and staging, particularly in cases where histopathological findings are inconclusive. These implications are consistent with the recommendations from the St. Gallen International Consensus Guidelines^1^ and previous studies emphasizing the prognostic value of Ki67.^15^,^28^

The strengths of our study include clearly defined inclusion and exclusion criteria, standardized immunohistochemical protocols, comprehensive lymph node sampling, and the simultaneous evaluation of multiple biomarkers, enhancing the internal validity and reliability of our results. Future research should focus on multi-center, prospective studies with larger, more diverse cohorts to validate our findings. Integrating molecular subtyping, genomic and transcriptomic profiling, and longitudinal follow-up will further clarify the prognostic and predictive utility of these markers and aid in the development of personalized therapeutic strategies.

***Limitations:***

First, it was a single-center retrospective analysis, which may limit the generalizability of the findings and introduce selection bias. Second, we did not evaluate dynamic changes in biomarker expression (Ki67, ER, PR, HER-2) before and after treatment, and therefore could not assess treatment-induced modulation of these indicators. Third, no follow-up or survival analyses were performed, so the associations between biomarker expression and long-term outcomes such as progression-free or overall survival remain unclear. Fourth, the overall sample size was relatively modest, and only three patients were classified as Stage IV, which may have reduced statistical power and increased the risk of type II error, particularly in subgroup analyses. Taken together, these factors may limit the robustness and external validity of our conclusions. Looking ahead, future studies will aim to address these limitations. We plan to conduct multi-center, prospective cohort investigations with larger and more diverse patient populations to validate the relationships between biomarker expression (Ki67, ER, PR, HER-2) and key clinical endpoints, including treatment response, survival, and recurrence. These studies will incorporate longitudinal follow-up to monitor dynamic changes in biomarker profiles before, during, and after therapy. Furthermore, integrating molecular and genomic profiling is expected to help elucidate the underlying biological mechanisms and support the development of predictive models to guide individualized therapy and risk stratification.

## CONCLUSION

Breast cancer patients exhibit characteristic alterations in Ki67, ER, and PR immunohistochemical expression, and higher Ki67 together with lower ER and PR labeling indices are closely associated with lymph node metastasis and more advanced clinical disease stage. Future multi-center prospective studies are warranted to validate these associations and to further clarify the clinical utility of combined biomarker assessment for predicting treatment response and guiding personalized management in breast cancer.

### Authors’ contributions:

**WQ:** Study design, literature search and manuscript writing.

**YN, ZW and FY:** Data collection, data analysis and interpretation. Critical review.

**WQ:** Manuscript revision and validation and is responsible for the integrity of the study.

All authors have read and approved the final manuscript.
